# Predictors of Executive Functions in Preschoolers: Findings From the SPLASHY Study

**DOI:** 10.3389/fpsyg.2018.02060

**Published:** 2018-10-29

**Authors:** Annina E. Zysset, Tanja H. Kakebeeke, Nadine Messerli-Bürgy, Andrea H. Meyer, Kerstin Stülb, Claudia S. Leeger-Aschmann, Einat A. Schmutz, Amar Arhab, Jardena J. Puder, Susi Kriemler, Simone Munsch, Oskar G. Jenni

**Affiliations:** ^1^Child Development Center, University Children’s Hospital Zurich, Zurich, Switzerland; ^2^Children’s Research Center, University Children’s Hospital Zurich, Zurich, Switzerland; ^3^Department of Clinical Psychology and Psychotherapy, University of Fribourg, Fribourg, Switzerland; ^4^Obstetric service, Lausanne University Hospital, Lausanne, Switzerland; ^5^Department of Psychology, University of Basel, Basel, Switzerland; ^6^Epidemiology, Biostatistics and Prevention Institute, University of Zurich, Zurich, Switzerland

**Keywords:** executive functions, predictors, preschoolers, motor skills, cognitive functioning, socio economic status, sex, SPLASHY

## Abstract

Executive functions (EFs) have been reported to play a crucial role in children’s development, affecting their academic achievement, health, and quality of life. This study examined individual and interpersonal predictors for EFs in 555 typically developing preschool children aged 2–6 years. Children were recruited from 84 child care centers in the German- and French-speaking parts of Switzerland within the Swiss Preschoolers’ Health Study (SPLASHY). A total of 20 potential predictors were assessed at the first measurement (T1). These included eight demographic/biological predictors, such as socioeconomic status, preterm birth, physical activity, and motor skills; six psychological predictors, such as hyperactivity, visual perception, and emotionality; and six interpersonal predictors, such as parenting style and stress, presence of siblings, and days spent in the child care center. The predictive value of these variables on EFs 1 year later (T2) was assessed using both standard multiple regression analysis and penalized regression to avoid overfitting due to the number of potential predictors. Female sex (β = 0.14), socio-economic status (β = 0.15), fine motor skills (β = 0.17), visual perception at T1 (β = 0.16), and EFs at T1 (β = 0.30) were all associated with EFs at T2, exhibiting small to medium effect sizes. All predictors together accounted for 31% of the variability in EFs. However, none of the interpersonal predictors were significant. Thus, we conclude that most of the factors that can predict EFs in preschool age are individual variables, and these tend to be more difficult to influence than interpersonal factors. In fact, children from families with low socio-economic status may be particularly vulnerable to poor EFs. Furthermore, encouraging fine motor skills early in life may support the development of EFs.

## Introduction

Executive functions (EFs) are control processes which regulate cognition and behavior (Miyake and Friedman, 2012). They enable goal-directed behavior, thus, they are particularly needed in situations, which involve planning and decision-making, and inhibition of inappropriate behavior. Several studies investigating the functional structure of EFs have identified three main components: inhibition, working memory, and cognitive flexibility (Miyake et al., 2000; Garon et al., 2008). The neural mechanisms that regulate executive processes are primarily located in the frontal cortex, and they develop with maturation of the individual (Miyake et al., 2000; Garon et al., 2008; Diamond, 2013). A central time window for the development of EFs is the first 5 years of life, because the main components develop in this period and lay the foundation for later EFs (Garon et al., 2008).

Executive functions have attracted increasing attention, because numerous associations have been found to many aspects of life from infancy through adulthood (e.g., see overview in Diamond, 2013): for instance, EFs in preschoolers have been shown to predict early academic performance (Cameron et al., 2012; Roebers et al., 2014; Mulder et al., 2017). Strong EFs in preschoolers have also been linked positively to later outcomes such as social interactions, job success, and marital harmony and negatively to externalizing behavioral problems, attentional deficit, and substance abuse (Eakin et al., 2004; Bailey, 2007; Young et al., 2009; Miyake and Friedman, 2012; Sawyer et al., 2015).

The widespread associations of EFs all demonstrate EFs’ importance. They also indicate the need to support EF development to facilitate the best possible academic achievement, health, and quality of life. Encouraging the development of EFs requires the identification of reliable predictors of EFs. Efforts can then be focused on those that can be trained or changed. Despite evidence that EFs are largely heritable (Engelhardt et al., 2015), some authors have reported factors such as positive parenting and physical activity as influencing EFs (Best, 2010; Hughes and Devine, 2017). Further influences have been assumed, but the cross-sectional design of previous studies did not allow causality to be confirmed. Hence, longitudinal studies are needed to clarify the causal direction of any such factors on EFs in early childhood. Following the approach of a socio-ecological model (Bronfenbrenner, 1995 in Kail, 2004; Bauman et al., 2012), the subsequent sections present a range of variables that previous studies have associated with EFs or found to predict later EFs. The sections deal in turn with individual demographic, biological, and psychological factors. They then deal with interpersonal factors such as parenting style, parenting stress, and the presence of siblings.

### Individual Demographic and Biological Factors

A study by Klenberg et al. (2001) reported that performance in EF tasks increases with age. Furthermore, these authors found that inhibition and impulse control mature earlier in girls than in boys. Children from families with higher socioeconomic status (SES) were found to perform better in EF tasks than children with low SES (Klenberg et al., 2001; Noble et al., 2005; Lawson et al., 2013). Noble et al. (2005) quantified the relationship between SES and EFs and reported that SES accounted for 15.3% of the variance in EF performance. Specifically, the educational level of parents explained most of this variance; no additional significant variance was explained by present occupation (score on the seven-point Hollingshead Occupation Status Scale) or family income (income-to-needs ratio). Furthermore, several studies have shown that preterm birth has an influence on EFs (Aarnoudse-Moens et al., 2012; Hagmann-von Arx et al., 2014; Wehrle et al., 2016). For instance, Wehrle et al. (2016) found that adolescents who were born very preterm (≤32 week) performed significantly lower in working memory, planning, and cognitive flexibility tasks compared to term-born children.

Two additional individual biological factors are motor skills and physical activity. EFs have repeatedly been found to be associated with motor skills (Livesey et al., 2006; Cameron et al., 2012; Gottwald et al., 2016). For example, Gottwald et al. (2016) postulated that EFs are ‘grounded in an infant’s developing ability to control and plan motor actions’ (p. 1601). These authors indeed found that better prospective motor control (measured as velocity of reaching for an object) in 18-month-olds was positively correlated cross-sectionally with better inhibition and working memory (*r* = 0.31 to −0.39). A study by Cameron et al. (2012) found that fine motor skills correlated with the head-toes-knee-shoulders EF task (*r* = 0.15) in 3-to-4-year-olds. In contrast, no association was found between planning in motor tasks and EFs (Tower-of-Hanoi-, Mosaic-, and d2-attention-endurance-task) in 3-to-10-year-old children in a study by Wunsch et al. (2016). One reason for this negative finding might be that the study used small groups (nine groups with on average 24 children), and planning skills were measured rather than motor skills *per se*. In a longitudinal study by Roebers et al. (2014), fine motor skills predicted only cognitive functioning, but not EFs (Backward Recall-, Fruit-Stroop-, and Cognitive Flexibility-task), in school-age children. Overall, there is strong evidence for an association between motor skills and EFs from cross-sectional studies, but the predictive value of motor skills on EFs is unclear.

Lastly, cumulative evidence exists that regular physical activity contributes positively to cognitive functioning and EFs (Dishman et al., 2006; Best, 2010; Niederer et al., 2011; Chaddock et al., 2012; Monti et al., 2012; Hillman and Schott, 2013). Three mechanisms by which physical activity might influence cognitive skills are assumed: “(1) increase in oxygen saturation based on an increased blood flow and angiogenesis, (2) increase in brain neurotransmitters like serotonin and norepinephrine facilitating information processing, and (3) regulation of neurotrophins such as different growth factors” (Ploughman, 2008 in Niederer et al., 2011, p. 2). It has been found that physical activity has a direct effect on the brain structures that are related to cognitive and executive processes (Chaddock et al., 2010, 2012).

### Individual Psychological Factors

A review article by Moriguchi (2014) suggested that social interactions might influence the development of EFs. There is evidence that better performance in EFs is associated with fewer behavioral problems, such as attention deficit disorder, hyperactivity, and conduct disorder (Hughes and Ensor, 2008; Young et al., 2009; Miyake and Friedman, 2012), thus, to clarify the causal direction longitudinal studies are necessary.

Another individual factor influencing the development of EFs is basic cognitive functions. However, it is not always easy to distinguish cognitive functions from EFs. To our knowledge, no definition has yet been agreed of the distinction between these two groups of functions. EFs are widely viewed as higher-order cognitive processes that are based on basic cognitive skills. Therefore, basic cognitive skills are related to EFs but do not account for the whole EF construct. The difference between cognitive skills and EFs becomes evident when comparing studies that have found a relation with only one of the two constructs. For example, motor skills predicted only cognitive functioning but not EFs in a study by Hughes and Devine (2017), while parenting influenced EFs but not cognitive functioning in another (Roebers et al., 2014).

### Interpersonal Factors

Siblings may promote children’s development of EFs. It is hypothesized that a child may observe and imitate EF skills by playing games with rules (e.g., “wait until it is your turn”), negotiation, and learning strategic games with siblings and thus learn such skills faster. Another factor may be that siblings react more negatively and directly to inappropriate behavior (e.g., not following the game rules) than parents and other adults (Cole and Mitchell, 2000). McAlister and Peterson (2006) studied the relationship between EFs, siblings, and theory of mind development. They found that the presence of at least one child-aged sibling in the household was associated with better performance in EFs but not with theory of mind performance (EFs were assessed by a route navigation tasks and a resisting instructions task). Thus, the presence of siblings might be associated with and even promote EFs.

Evidence is accumulating that parenting style also has an effect on EFs. A study by Hughes and Devine (2017) showed that adverse parenting (negative affect, criticism, control) is negatively associated (β = −0.23) with EFs (Stroop-task, Dimensional Change Card Sort-task, and working memory). In contrast, positive parenting (scaffolding/supporting) showed a positive association (β = 0.19). However, neither parenting style showed a significant association with basic cognitive ability (measured with object assembling). In this 13-month longitudinal study by Hughes and Devine (2017) with 117 3-to-4-year-old children, neither the EFs of parents nor their education showed any effect on children’s EFs. Another study by Blair et al. (2014) found that higher parental sensitivity and responsiveness at the age of 3 years was associated with higher child EFs at age of 5 years (EFs were assessed by a validated battery of 6 EF tasks measuring inhibitory control, working memory, and attention shifting). In contrast, another study found no association between EFs and parenting behavior; this study (Röthlisberger et al., 2010) measured the quantity of time that parents spent engaging with physical and learning activities, playing games, and talking with their child. EFs were measured by working memory, cognitive flexibility and a Stroop-task. Thus, qualitative aspects of the relationship between parents and children, such as being supportive and responsive seem mainly to have an effect on EFs rather than simple quantity of time. The supportive behavior of teachers can also have a positive effect on EF performance, especially when the parent–child relationship is conflictual (Vandenbroucke et al., 2017). Accordingly, institutions such as child care centers may be beneficial for the development of EFs, both through supporting relationships with child care educators and through social interactions with same-age children, which are likely to encourage following rules and appropriate behavior similarly to interaction with siblings.

In sum, many studies have been conducted on EFs, and a diverse range of associations have been identified. However, little evidence for factors predicting EFs is available from longitudinal studies. Furthermore, most studies have focused on only one predictor, not a comprehensive set of potential predictors, rendering direct comparison of magnitudes of influence on EFs infeasible. Finally, studies in young children are still scarce compared to studies involving school-age children and adolescents.

The aim of the present study was to examine potential predictors of EFs in typically developing preschoolers, including demographic, biological, psychological, and interpersonal variables. In contrast to previous studies, we investigated possible indicators from all domains affecting EFs. We selected variables used in previous research, following the approach of a socio-ecological model. Based on this analysis, we aimed to identify the factors most crucial to promoting EFs. We used a model of penalized regression that allowed variable selection and avoided overfitting due to the number of predictors tested simultaneously.

## Materials and Methods

### Participants

Data were drawn from the Swiss Preschoolers’ Health Study (SPLASHY, ISRCTN41045021), which is a multi-site, prospective cohort study of healthy children at preschool age. The sample for the current analysis thus consisted of 555 children (52.8% boys) between 2 and 6 years of age at their first measurement (mean = 3.9, *SD* = 0.7). Children were recruited from 84 child care centers in five cantons of Switzerland (Aargau, Bern, Fribourg, Vaud, and Zürich). These cantons together comprised 50% of the Swiss population in 2013. Recruitment of child care centers was stratified in four levels: urban and rural communities with high SES (above-average) and low SES (below-average), each based on the prevalence of child care centers in the communities. The detailed study design and overall objectives have been described in a methodological paper (Messerli-Burgy et al., 2016).

A total of 476 children participated in the first baseline assessment in 2014. In the follow-up assessment 1 year later, 382 of these children participated again (20% dropped -out), and 79 new children were tested for baseline (total 555 children). The same study team conducted data collection at baseline and follow-up in parallel at all study sites. While children recruited in 2014 (*n* = 476) could participate in the follow-up assessment, those recruited in 2015 (*n* = 79) underwent only baseline assessment. Baseline (T1) and follow-up (T2) data are used in this study. The study was approved by all local ethical committees (No. 338/13 for the Ethical Committee of the Canton of Vaud as the main ethical committee) and is in accordance with the Declaration of Helsinki. Parents provided written informed consent, and children provided oral consent.

### Procedure

Subjects were tested in their own child care centers on three afternoons: on the first afternoon, a motor test was performed, and body composition was measured; on the second afternoon, self-regulation and executive and cognitive functioning was assessed; and on the third afternoon, a stress reaction test was executed. This last test was not included in the current analysis. Each child was tested individually, without the presence of his/her parents. All examiners were trained, and quality checks were performed periodically. In addition to the testing afternoons, each child wore an accelerometer for an entire week. Parents completed a questionnaire on general health (including anamneses of the child, demographical and environmental information of the family) and a questionnaires on psychological well-being (including characteristics of the child and parenting style). All questionnaires could be completed online or on paper.

### Measures

#### Predictors (T1)

Eight demographic and biological variables were included. Information about *sex, prematurity* (<37 weeks: yes/no), and *SES* were drawn from the general health questionnaire, which was constructed for the study by the research team. Sex was coded as zero for male and one for female. The SES of the family was calculated by coding the occupational status of both parents and transforming this into an International Socio-Economic Index (ISEI-08) value (Ganzeboom, 2010). Scores for this index can range from 16 for an unskilled worker to 90 for a judge. The maximal SES was then determined by the selection of the highest of the parental ISEI values. *Body fat* was measured by skinfold thickness of the triceps, biceps, subscapular, and suprailiac crest using standard procedures (Lohman et al., 1988). The sum of all four skinfolds was calculated. *Motor skills* included fine motor skills, pure motor skills, and associated movements; these were assessed using the Zürich Neuromotor Assessment 3-5 (ZNA 3-5; Kakebeeke et al., 2012, 2013). Associated movements are involuntary movements that accompany the voluntary movement of a motor task and are assumed to indicate immaturity of the motor system. *Physical activity* was recorded objectively using a hip-worn accelerometer that measured tri-axial acceleration (wGT3X-BT, ActiGraph, Pensacola, FL, United States) for seven consecutive days. Accelerometer data was sampled at a frequency of 30 Hz, downloaded in 3-s epochs, aggregated to 15-s epochs, and expressed as accelerometry counts per min averaged over the recording time and as time spent at various activity intensities. For this analysis, we used only physical activity spent in moderate to vigorous intensity. Cut-points were based on findings by Pate et al. (2006) for moderate to vigorous intensity (≥420 counts per 15 s).

Six psychological, cognitive and emotional variables were included. *Hyperactivity, problems with peers*, and *prosocial behavior* of the child were rated online by the parents using the parental version of the Strength and Difficulties Questionnaire (SDQ; Goodman, 2001). The subscales achieved reliability scores of α = 0.69 (hyperactivity/inattention), α = 0.49 (peer problems), and α = 0.66 (prosocial behavior). *Emotionality* (α = 0.71) was also rated online by the parents using the Emotionality Activity Sociability Temperament Survey (EAS; Buss and Plomin, 1984). *Visual perception* (IDS-P; Grob et al., 2013) was assessed as estimation of cognitive skills The goal of the tasks was to order eight times five cards with pencils according to different sizes of the pencils on each card, from the smallest to the biggest. For each card put in the right position one point was given. Per sub-item a maximal score of five points was possible. For all eight sub-items a total score of 40 points could be reached. This sensory discrimination ability task has been found to correlate highly with general intelligence (*r* = 0.78–0.96) (Spearman, 1904; Meyer et al., 2010). *EFs at T1* were included in the predictor list, measured analogously to EFs (T2) by calculating a mean of selective attention, self-regulation, and visuo-spatial working memory (see description of outcome measures).

Six interpersonal variables were included. *Parenting stress* was gathered online by a self-report using the Parental Stress Scale (PSS; Berry and Jones, 1995). Internal consistency in our sample was α = 0.80. *Parenting style* was collected online by the Alabama Parenting Questionnaire (APQ; Reichle and Franiek, 2009). This analysis included the subscales *positive parenting* and *inconsistent parenting*, which exhibited internal consistencies of α = 0.74 and α = 0.71 respectively. In the general health questionnaire, we asked whether at least one *sibling* (≤18 years) of the child lived in the household. Information was collected about the *time that the child spent outdoors* (min/day), and number of days that the child visited the *child care center* (half days/week).

All variables were z-standardized to provide the same units of measurement for the analysis.

#### Outcome: Executive Functioning (T2)

*Selective attention*, from the Intelligence and Development Scales – Preschool (IDS-P; Grob et al., 2013) is the child version of the d2-attention-endurance test (Brickenkamp, 1994). The task requires selective attention, inhibition, and speed of processing to achieve good results. The task can be used to measure the inhibition component of EFs. The child had to sort cards showing ducks with yellow or white beaks. The goal of the task is to separate the cards into two piles, one of each type of card, as quickly as possible. Some cards also showed a yellow sun, which the child had to ignore. As many cards as possible had to be sorted within 90 s. The task was scored immediately during the test. The score for each child was calculated as the total number of sorted cards minus the number of incorrectly sorted cards. A maximum score of 72 points was possible.

*Self-regulation* was measured with the statue subtest of the Neuropsychological Assessment for Children (NEPSY; Korkman et al., 1998). The statue test is an indicator of motor inhibition and resistance to interference from distractors. The task can be used to measure the inhibition component of EFs. The child was asked to maintain the position of a statue holding a flag with closed eyes for 75 s. The child was instructed to avoid moving, opening eyes, or speaking until the experimenter finished the test. During the test, the experimenter made several noises intended to distract the child. The task was videotaped, and the clips were coded by experienced psychologists for movements of body parts and facial reactions. Interrater reliability achieved α = 0.99. Children with no movement, no eye opening, and no vocalization achieve a maximum of two points per 5-s interval; for a single inappropriate response, they received one point, and they received zero points for several inappropriate responses. A total of 30 points was possible.

*Visuo-spatial working memory* (IDS-P; Grob et al., 2013) requires focusing on and remembering of geometric form while ignoring color. The task can be used to measure the working memory/updating component of EFs. The child was instructed to remember colored geometric figures, presented on a page, and recognize them afterward on a new page with other geometric figures. The relevant cue was the geometric shape; the color had to be ignored. The number of items to remember increased from one to four during the task. One point was given for remembering all figures. A half-point was given for remembering some of the figures (e.g., two out of three), and zero points were given for not remembering or remembering the wrong figures. A total score of 10 points was possible.

The outcome EFs (T2) were calculated as a mean of all three tasks described above. All scores for these tasks were transformed into standard deviation scores (SDS) and adjusted for age, with positive values corresponding to above-average performance and negative values to below-average performance.

#### Statistical Analyses

Statistical analyses were performed using R version 3.3.1 (R Foundation for Statistical Computing, Vienna, Austria), including the R packages *glmnet* and *caret* for the lasso model (Friedman et al., 2010; Kuhn et al., 2016) and *mice* for multiple imputation (van Buuren and Groothuis-Oudshoorn, 2011). Descriptive statistics were calculated by means ± standard deviations for continuous variables and percentages for categorical variables (Table 1). To investigate the relationship between predictors at T1 and EFs at T2, we applied two different regression models. We first used a multiple regression model, which included the entire list of predictors to be tested (Table 1). Because multiple regression models regularly suffer from overfitting, leading to models with low predictive accuracy, we also used a variable selection procedure, the least absolute shrinkage and selection operator (lasso), as a second model (Hastie et al., 2009).

**Table 1:** Descriptive statistics of predictors tested.

	*n*	*M or %*	*SD*	Range
**Individual factors**				
**Demographic and biological variables**				
Sex (% boys)	555	52.8	–	1/2
SES (ISEI score)	520	62.9	15.5	17 to 89
Born preterm (% yes)	516	7.6	–	0/1
Body fat (mm)^1^	495	26.0	5.5	14.6 to 51.0
Fine motor skills (SDS)	495	0.1	1.0	−3.1 to 3.4
Pure motor (SDS)	461	0.1	1.2	−4.6 to 3.8
Associated movements (SDS)	429	−0.1	1.0	−3.3 to 3.0
Moderate to vigorous PA (min/day)	505	92.0	29.7	25.9 to 206.5
**Psychological variables**				
Hyperactivity/inattention^2^	510	3.2	2.0	0 to 10
Peer problems^2^	511	1.2	1.4	0 to 6
Prosocial behavior^2^	511	7.7	1.7	2 to 10
Emotionality temperament^3^	511	2.8	0.7	1 to 5
Visual perception (SDS)	513	0.0	1.0	−2.7 to 2.5
EFs (T1, SDS)^4^	449	0.0	0.7	−2.2 to 1.6
**Interpersonal factors**				
Parenting stress^5^	511	37.4	7.4	20 to 68
Positive parenting^6^	511	4.5	0.4	3.0 to 5.0
Inconsistent parenting^6^	511	2.5	0.5	1.0 to 4.2
Siblings (% yes)	552	67.8	–	0/1
Time outdoors (min/day)	507	144.1	87.5	0.0 to 480.0
Half days in childcare	548	5.5	2.6	0 to 10

*Where no unit of measurement is indicated, scores refer to the corresponding questionnaire scale. ^1^Body fat is the sum of four skinfolds; ^2^Strength and Difficulties Questionnaire; ^3^Emotionality Activity Sociability Temperament Survey; ^4^EFs T1 is the mean of selective attention, self-regulation, and visuo-spatial working memory at T1; ^5^Parental Stress Scale; ^6^Alabama Parenting Questionnaire.*

In the lasso model, coefficients are shrunk by implying a penalty term to the estimated sum of squares of the residuals when fitting the model (Hastie et al., 2009). Therefore, lasso models are slightly more biased than multiple regression models, but they often show strongly increased predictive accuracy. Hence, predictors whose coefficients from penalized regression have not been shrunk to zero are more likely to be predictive when replicating the study. Simply put, the lasso technique removes unimportant predictors from the model by setting their coefficients to zero while the more relevant correlate variables remain. All variables were standardized prior to analysis for the lasso model. Since no tests of significance are available for the lasso method, no *p*-values are reported.

Multicollinearity was tested among the predictors involved in the analysis but presented no issue here (variance inflation factors ranging from 1.06 to 1.38). The data contained missing values; see Table 1 for data available for each predictor. Missing values were substituted using multiple imputation techniques. Prior to any analysis, missing values were repeatedly (i.e., 50 times) imputed using chained equations as implemented in the *mice* R package (van Buuren and Groothuis-Oudshoorn, 2011). Each imputation creates a different dataset in which estimated values replace missing values. Regression models were run 50 times using each of the complete datasets, and results were then pooled across the 50 datasets. To our knowledge, no technique yet exists to combine lasso-based results from several data files. Therefore, we determined the importance of each potential predictor by calculating the mean and standard deviation of each lasso coefficient across all 50 data files. Finally, mean standardized lasso coefficients are presented.

To facilitate comparison of results, we focus on the multiple regression model and add the lasso results as auxiliary. As Supplementary Material, we present results of single regression models for each predictor, using the original dataset containing only observed data. We mention the main concordances and discordances between the multiple regression model, the lasso model, and single regression models in the text.

## Results

Descriptive statistics of predictors to be tested are shown in Table 1, which contains mean and standard deviation or percentage, range, and the number of children with data available for each variable.

Results of the multiple regression model are presented in Table 2. In this analysis, all predictors together accounted for 31% of the variability in EFs at T2. As coefficients can be interpreted analogously to correlation coefficients (0.1 small, 0.3 moderate, and >0.5 large; Cohen, 1992), the effect sizes were all between small and medium. The largest effect size was found for EFs T1 (β = 0.30). Other significant predictors were sex (β = 0.14), SES (β = 0.15), and fine motor skills (β = 0.17) and visual perception (β = 0.16).

**Table 2:** Tested predictors of executive functions multiple regression model.

Predictors	β	95% CI	*p*-Value
**Individual factors**			
**Demographic and biological variables**			
Sex^1^	0.14	0.04, 0.23	0.00
SES	0.15	0.05, 0.24	0.00
Born preterm	−0.05	−0.15, 0.05	0.33
Body fat	−0.05	−0.14, 0.04	0.29
Fine motor skills	0.17	0.06, 0.28	0.00
Pure motor	0.08	−0.04, 0.19	0.19
Associated movements	0.00	−0.11, 0.11	0.98
Moderate to vigorous PA	0.03	−0.05, 0.13	0.35
**Psychological variables**			
Hyperactivity/inattention	−0.07	−0.16, 0.03	0.15
Peer problems	0.06	−0.04, 0.15	0.24
Prosocial behavior	−0.04	−0.13, 0.05	0.40
Emotionality temperament	0.01	−0.08, 0.10	0.82
Visual perception	0.16	0.05, 0.26	0.00
EFs (T1)	0.30	0.19, 0.41	0.00
**Interpersonal factors**			
**Family**			
Parenting stress	−0.01	−0.10, 0.09	0.88
Positive parenting	0.01	−0.08, 0.10	0.83
Inconsistent parenting	0.01	−0.07, 0.10	0.77
Siblings	0.01	−0.08, 0.09	0.84
Time outdoors	0.04	−0.04, 0.12	0.33
Half days in childcare	−0.06	−0.16, 0.04	0.25

*Coefficients are based on multiple regression model. All variables were standardized. ^1^Coded 0 = male/1 = female.*

Table 3 contains the shrunk coefficients resulting from the lasso model. The cross-validated lasso model accounted for 32% of the variability in EFs, while the cross-validated standard multiple regression model accounted for 31% of the variability. Compared to the significant predictors in the multiple regression model (Table 2), the coefficients in the lasso were only slightly shrunk (3–14%), suggesting that the multiple regression model was only slightly overfitted. Compared to the non-significant predictors in the multiple regression model, the lasso coefficients were generally more shrunk (0–100%) and often set to zero.

**Table 3:** Tested predictors of executive functions lasso model.

Predictors	Mean (β)
**Individual factors**	
**Demographic and biological variables**	
Sex	0.13
SES	0.13
Born preterm	−0.03
Body fat	−0.02
Fine motor skills	0.16
Pure motor	0.05
Associated movements	0.01
Moderate to vigorous PA	0.01
**Psychological variables**	
Hyperactivity/inattention	−0.03
Peer problems	0.03
Prosocial behavior	−0.01
Emotionality temperament	0.00
Visual perception	0.13
EFs (T1)	0.29
**Interpersonal factors**	
**Family**	
Parenting stress	0.00
Positive parenting	0.00
Inconsistent parenting	0.00
Siblings	0.00
Time outdoors	0.02
Half days in childcare	−0.04

*Coefficients are shrunken and based on a lasso model. For the lasso method, no tests of significance are available yet, so no p-values are reported.*

The results of the univariate regression models testing one predictor at a time without multiple imputation are shown in the Supplementary Table 1. The comparison between Tables 2–4 reveals the benefits of our methodological approach. While univariate regression analyses support our prior results, it also reveals additional significant predictors, which disappear in the lasso model, and thus, are not likely to be predictive.

## Discussion

The aim of this study was to identify possible predictors of EFs in preschool-age children. We selected a broad spectrum of individual demographic, biological, and interpersonal factors from previous published evidence, measured these in a cohort of preschool children, and analyzed their predictive value on EFs 1 year later. The results show that the factors that significantly predicted EFs are all individual ones. We found that female sex, higher SES, better fine motor skills, better visual perception, and better EFs at the first measurement were all predictors of higher EFs 1 year later. Interpersonal factors, such as parenting style, parenting stress, the presence of siblings, or amount of days that the child visited a child care center, did not predict EFs.

Our results showed that female sex and SES, despite small effect sizes, were among the most important determinants of EFs. Sex predicting EFs is in line with previous studies in preschoolers that found an association between sex and EFs, with girls outperforming boys (Klenberg et al., 2001). The plausible explanation is that girls mature earlier (Lim et al., 2015) and therefore EF development is more advanced in girls. Lim et al. (2015) found that the neuronal reorganization that makes information processing more efficient occurs earlier in girls than in boys.

Furthermore, we found that SES predicted EFs. Previous studies had already shown an association between SES and EFs. For example, in the cross-sectional study by Noble et al. (2005), SES was associated with EFs with a moderately large effect size. Although in our study SES predicted EFs with only a small effect size, our result supports the role of SES in EF development. The mechanism underlying this association is not yet fully understood. However, it has been noted that SES involves more than the variability in occupation, income, and education, which are the characteristics by which it is defined (Noble et al., 2005). Such factors as home environment, childhood experience, stimulation in childhood, health care access, early life stress, and neighborhood conditions are closely linked with SES and influence the development of children (Noble et al., 2005; Lupien et al., 2007, 2009; Moriguchi, 2014). Another essential link has been reported between SES, language skills, and EFs; In one previous study, SES and language skills both predicted EFs, but, SES explained no additional variance in EFs when language skills were controlled for (Noble et al., 2005). In fact, language skills might play a role in EF development via the self-regulatory function of language. For instance, language skills are needed in several EF tasks, and working memory performance depends on strategies that often involve verbal rehearsal (Hughes and Graham, 2002). No language skills where assessed in this study, so the causality path hypothesized here cannot be tested. However, perhaps SES and its related factors primarily influence the language skills that then drive EF performance.

Contrary to expectations and to previous findings (Aarnoudse-Moens et al., 2012; Hagmann-von Arx et al., 2014; Wehrle et al., 2016), being born preterm was not significant in predicting EFs. The predictor variable preterm born revealed a negative association with EF performance in the single regression analysis, but the effect disappeared in the multiple regression model and the lasso model. The reason of no effect might be that, although 8% in our sample were born preterm (≤37 weeks), few children (1.4%) were born very preterm (<32 weeks). Due to the low number of preterm-born children, we decided not to split them into even smaller groups, but the mingling of preterm-born and very preterm-born children and the generally low sample size of preterm-born might be the reason for the absence of a significant predictive effect. Alternatively, preterm birth may have no predictive value after controlling for all the predictors included in the current study.

In line with previous results, fine motor skills predicted EFs. However, pure motor skills and associated movement showed no predictive effect. Pure motor skills are largely independent of experience and reflect motor speed. Associated movements are involuntary movements caused by failure of motor inhibition in the contralateral body side during motor tasks; these can be interpreted as indicators of the degree of maturation of the motor system. Hence, motor speed and motor inhibition seem not to predict EFs. In contrast, fine motor skills predicted EFs with a small effect size in both the multiple regression and the lasso model and thus can be assumed to be a reliable predictor of EFs.

The last biological predictor to be examined was moderate to vigorous physical activity. Although positive effects of physical activity on EFs and cognitive functions in general have repeatedly been reported (Chaddock et al., 2012; Hillman and Schott, 2013), no effect was found in this study. Best (2010) reported that physical activity that involves greater cognitive engagement in particular leads to increased EFs. He also proposed that ‘EF may be more sensitive to aerobic exercise at one developmental time point than at another, and one EF component may be more sensitive to acute aerobic exercise than another’ (Best, 2010, p. 6). Thus, in 2-to-6-year olds, when EFs are developing, an effect of physical activity might not yet be ascertainable. We coded the amount and intensity level of physical activity, but not what the children were actually doing at each stage. Moreover, the children in our sample met the recommended guidelines for physical activity (minimum 180 min/day total physical activity and minimum 60 min/day moderate to vigorous physical activity; Leeger-Aschmann et al., 2016). Thus, we had a generally physically active and healthy sample. These factors might explain why no effect of physical activity could be found in our sample.

Child characteristics and temperament did not predict EFs. Hyperactivity was not predictive in the main analysis, but in the single analysis hyperactivity accounted for 2% variability in EFs. It is assumed that EFs and hyperactivity/inattention are linked, since lack of inhibition is likely to lead to hyperactivity and inattention. However, these two characteristics may influence test performance; being unable to focus and concentrate on the tasks, the child might score lower on EF tasks. This interaction should be kept in mind when testing EFs. Again, no effect was observed in our sample. Prosocial behavior was hypothesized to have a positive influence on EFs through training of inhibition skills, but no effect was apparent. Thus, child temperament and characteristics did not predict EFs. Our sample exhibited low scores for hyperactivity, peer problems, and emotionality and high scores on prosocial behavior. Of the psychological variables that were examined, only visual perception and EFs at the first measurement predicted EFs 1 year later. Visual perception predicted EFs with a small effect size. As stated in the introduction, the distinction between basic cognitive skills and EFs is not always clear. This result indicates that basic cognitive skills as sensory discrimination ability can have an independent effect on EFs 1 year later regardless of EFs at baseline. EFs at the first measurement predicted EFs 1 year later with only a moderate effect size, indicating that EFs might not be very stable at this age.

Unexpectedly, we found no effect of parenting on EFs. Previous studies have provided evidence that quality of parenting does influence EFs (Blair et al., 2014; Hughes and Devine, 2017; Vandenbroucke et al., 2017), but we found no effect of parenting stress, positive parenting, or inconsistent parenting. In the lasso model, all these coefficients shrank to zero, indicating a very low probability that parenting factors influence EFs. Once again, this may be because parents in our sample did not report high levels of parenting stress (37.4) or negative parenting (2.5) and showed a normal level of positive parenting (4.5). All scores corresponded to the norm population [norm of parenting stress: 37.18 (Stadelmann et al., 2010); norm inconsistent parenting: 2.47; norm positive parenting: 4.25 (Reichle and Franiek, 2009)]. Parenting variables are based on self-reporting, which can be biased by social desirability. However, previously reported parenting effects have generally been small. While an association between parenting and SES may exist (as discussed above), SES was more important than parenting style for predicting EFs in our study.

No effect was found for attending a child care center. We hypothesized that more days at the child care center would provide the child with greater benefit from social interaction with same-age children, positive support from child care educators, and promoted physical activities. Similarly, no effect was found for having siblings or time spent outdoors. Finally, no predictive effect was found from parenting style and parenting stress.

An explanation for the differences to other studies might be the tasks used to measure EF in the current study. In fact, in all reviewed studies EF was measured with different tasks. Thus, the age of the children and, thus, the age-appropriate tasks might have influenced our findings. Furthermore, it maybe that separate models for the three EF tasks would lead to different results, as done for example in the study by Niederer et al. (2011), although we would expect that the same factors remain significant. We believe, however, that the results would not change fundamentally.

A limitation of SPLASHY is that we have only two measurement points within a relatively short period, just 1 year. Further measurement points later in development would be desirable to define predictors of EFs more precisely. Some factors which did not show an effect on EFs in preschool age might do so later. Furthermore, the coverage of this study is limited to typically developing children with minimal risk characteristics and psychopathologies. Notably, we could not cover all possible predictors of EFs. Certainly, there would be more factors to include such as nutrition or sleep behavior. Another limitation is the low internal reliability of the peer problems subscale. Thus, the results regarding this predictor have to be interpreted with caution. However, a clear strength of SPLASHY is that we examined a large sample representative of a typically developing community-based population of children within different social cultural regions of Switzerland and containing urban and rural communities with high and low SES. Another strength is that we compared different statistical analyses to prevent bias in the results.

Engelhardt et al. (2015) stated that, in contrast to adulthood, when the non-genetic variance from factors not based on biology, is rather small, the non-genetic variance of EFs in childhood is not yet clear. The current study contributes to answering this question. Our results indicate that genetic determinants may play an important role even at preschool age, because all predictors from different domains together accounted only for 32–36% of the variability in EFs, and among these, sex is also genetically determined. Further, consensus has yet to be reached on the extent to which motor skills and cognitive functioning are dependent on predisposition and environment. Thus, a part of the variance our predictors could explain in EFs is genetically determined, with the consequence that the definitely non-genetic variance is even lower. Another aspect that should be investigated further is the relation between fine motor skills, cognitive functioning, and EFs. This study found that fine motor skills and visual perception predicted EFs, but other studies also found evidence that EFs are needed for complex motor and cognitive tasks and so might themselves be a source of motor and cognitive performance (Livesey et al., 2006; Roebers and Kauer, 2009).

Overall, we had to accept that not so many factors in the preschool age period that predict EFs can be influenced by parents and professionals. Variables that might be influenced, such as parenting style, physical activity, time spent outdoors, and days in a child care center, did not predict EFs. Given our result, encouraging motor skills could be beneficial to EFs. Additionally, high SES was positively associated with higher EFs. Thus, children coming from families with low SES might be a vulnerable group regarding EF development, so this group would likely benefit most from interventions. Conversely, promotion of EFs might be of little use and even unnecessary for children from middle and high SES families. An alternative explanation is that families with high SES may already stimulate the development of EFs through factors that we have not directly investigated. As mentioned above, diverse factors such as home environment, childhood experience, stimulation in childhood, health care access, early life stress, and neighborhood conditions are all linked to SES and may all influence the development of EFs. Further studies should focus on a more precise investigation of the influence of the factors linked to SES on EFs. Finally, EFs in the age range we assessed are only starting to develop and are not yet stable. This instability might have contributed to the low predictive value of the factors we studied. However, this also implies that preschool age is a sensible age at which to support EF development, because the main components that lay the foundation for later EFs are still developing during this time. Thus, more longitudinal studies, including later time points in development such as school age, are needed in the future.

## Author Contributions

AZ wrote the manuscript and analyzed the data. AZ, TK, NM-B, KS, CL-A, ES, and AA recruited, tested, and collected the data. AM assisted in statistical data analysis. SK, SM, JP, and OJ conceived and designed the SPLASHY study. All authors reviewed the manuscript and approved the final version for submission.

## Conflict of Interest Statement

The authors declare that the research was conducted in the absence of any commercial or financial relationships that could be construed as a potential conflict of interest.

## References

[B1] Aarnoudse-Moens C. S. H. Duivenvoorden H. J. Weisglas-Kuperus N. Van Goudoever J. B. Oosterlaan J. (2012). The profile of executive function in very preterm children at 4 to 12 years. *Dev. Med. Child Neurol.* 54 247–253. 10.1111/j.1469-8749.2011.04150.x 22126188

[B2] Bailey C. E. (2007). Cognitive accuracy and intelligent executive function in the brain and in business. *Ann. N. Y. Acad. Sci.* 1118 122–141. 10.1196/annals.1412.011 17717092

[B3] Bauman A. E. Reis R. S. Sallis J. F. Wells J. C. Loos R. J. F. Martin B. W. (2012). Correlates of physical activity: why are some people physically active and others not? *Lancet* 380 258–271. 10.1016/S0140-6736(12)60735-1 22818938

[B4] Berry J. O. Jones W. H. (1995). The parental stress scale - initial psychometric evidence. *J. Soc. Pers. Relat.* 12 463–472. 10.1177/0265407595123009

[B5] Best J. (2010). Effects of physical activity on children’s executive function: contributions of experimental research on aerobic exercise. *Dev. Rev.* 30 331–351. 10.1016/j.dr.2010.08.001 21818169 PMC3147174

[B6] Blair C. Raver C. C. Berry D. J. Family Life Project Investigators. (2014). Two approaches to estimating the effect of parenting on the development of executive function in early childhood. *Dev. Psychol.* 50 554–565. 10.1037/a0033647 23834294 PMC4682354

[B7] Brickenkamp R. (1994). *Test d2: Aufmerksamkeits-Belastungs-Test.* Göttingen: Hogrefe Verlag für Psychologie.

[B8] Bronfenbrenner U. (1995). “Developmental ecology through space and time: a future perspective” in *Examining Lives in Context: Perspectives on the Ecology of Human Development*, eds Moen P. Elder G. H. Jr. Lüscher K. (Washington, DC: American Psychological Association), 619–647. 10.1037/10176-018

[B9] Buss A. H. Plomin R. (1984). *Temperament: Early Developing Personality Traits.* Hillsdale, NJ: Erlbaum.

[B10] Cameron C. E. Brock L. L. Murrah W. M. Bell L. H. Worzalla S. L. Grissmer D. (2012). Fine motor skills and executive function both contribute to kindergarten achievement. *Child Dev.* 83 1229–1244. 10.1111/j.1467-8624.2012.01768.x 22537276 PMC3399936

[B11] Chaddock L. Erickson K. I. Prakash R. S. Kim J. S. Voss M. W. Vanpatter M. (2010). A neuroimaging investigation of the association between aerobic fitness, hippocampal volume, and memory performance in preadolescent children. *Brain Res.* 1358 172–183. 10.1016/j.brainres.2010.08.049 20735996 PMC3953557

[B12] Chaddock L. Hillman C. H. Pontifex M. B. Johnson C. R. Raine L. B. Kramer A. F. (2012). Childhood aerobic fitness predicts cognitive performance one year later. *J. Sports Sci.* 30 421–430. 10.1080/02640414.2011.647706 22260155

[B13] Cohen J. (1992). A power primer. *Psychol. Bull.* 112 155–159. 10.1037/0033-2909.112.1.155 19565683

[B14] Cole K. Mitchell P. (2000). Siblings in the development of executive control and a theory of mind. *Br. J. Dev. Psychol.* 18 279–295. 10.1348/026151000165698 21418062

[B15] Diamond A. (2013). Executive functions. *Annu. Rev. Psychol.* 64 135–168. 10.1146/annurev-psych-113011-143750 23020641 PMC4084861

[B16] Dishman R. K. Berthoud H. R. Booth F. W. Cotman C. W. Edgerton V. R. Fleshner M. R. (2006). Neurobiology of exercise. *Obesity* 14 345–356. 10.1038/Oby.2006.46 16648603

[B17] Eakin L. Minde K. Hechtman L. Ochs E. Krane E. Bouffard R. (2004). The marital and family functioning of adults with ADHD and their spouses. *J. Atten. Disord.* 8 1–10. 10.1177/108705470400800101 15669597

[B18] Engelhardt L. E. Briley D. A. Mann F. D. Harden K. P. Tucker-Drob E. M. (2015). Genes unite executive functions in childhood. *Psychol. Sci.* 26 1151–1163. 10.1177/0956797615577209 26246520 PMC4530525

[B19] Friedman J. Hastie T. Tibshirani R. (2010). Regularization paths for generalized linear models via coordinate descent. *J. Stat. Softw.* 33 1–22. 10.18637/jss.v033.i01 20808728 PMC2929880

[B20] Ganzeboom H. B. G. (2010). A new international socio-economic index [ISEI] of occupational status for the international standard classification of occupation 2008 [ISCO-08] constructed with data from the ISSP 2002-2007, with an analysis of quality of occupational measurement in ISSP. *Paper Presented at the Annual Conference of International Social Survey Programme*, Lisbon.

[B21] Garon N. Bryson S. E. Smith I. M. (2008). Executive function in preschoolers: a review using an integrative framework. *Psychol. Bull.* 134 31–60. 10.1037/0033-2909.134.1.31 18193994

[B22] Goodman R. (2001). Psychometric properties of the strengths and difficulties questionnaire. *J. Am. Acad. Child Adolesc. Psychiatry* 40 1337–1345. 10.1097/00004583-200111000-00015 11699809

[B23] Gottwald J. M. Achermann S. Marciszko C. Lindskog M. Gredebäck G. (2016). An embodied account of early executive-function development. *Psychol. Sci.* 27 1600–1610. 10.1177/0956797616667447 27765900 PMC5154392

[B24] Grob A. Reimann G. Gut J. Frischknecht M.-C. (2013). *IDS-P: Intelligence and Development Scales - Preschool : Intelligenz- und Entwicklungsskalen für das Vorschulalter.* Göttingen: Hogrefe.

[B25] Hagmann-von Arx P. Perkinson-Gloor N. Brand S. Albert D. Holsboer-Trachsler E. Grob A. (2014). In school-age children who were born very preterm sleep efficiency is associated with cognitive function. *Neuropsychobiology* 70 244–252. 10.1159/000369026 25720547

[B26] Hastie T. Tibshirani R. Friedman J. (2009). *The Elements of Statistical Learning : Data Mining, Inference, and Prediction.* New York, NY: Springer 10.1007/978-0-387-84858-7

[B27] Hillman C. H. Schott N. (2013). Fitness and cognitive performance in childhood. *Z. Sportpsychol.* 20 33–41. 10.1026/1612-5010/A000085

[B28] Hughes C. Devine R. T. (2017). For better or for worse? positive and negative parental influences on young children’s executive function. *Child Dev.* 10.1111/cdev.12915 [Epub ahead of print]. 28800148

[B29] Hughes C. Ensor R. (2008). Does executive function matter for preschoolers’ problem behaviors? *J. Abnorm. Child Psychol.* 36 1–14. 10.1007/s10802-007-9107-6 17914667

[B30] Hughes C. Graham A. (2002). Measuring executive functions in childhood: Problems and solutions? *Child Adolesc. Ment. Health* 7 131–142. 10.1111/1475-3588.00024

[B31] Kail R. V. (2004). *The Science of Child Development. Children and their development.* London: Pearson Education, 2–25.

[B32] Kakebeeke T. H. Caflisch J. Chaouch A. Rousson V. Largo R. H. Jenni O. G. (2013). Neuromotor development in children. Part 3: Motor performance in 3-to 5-year-olds. *Dev. Med. Child Neurol.* 55 248–256. 10.1111/Dmcn.12034 23278183

[B33] Kakebeeke T. H. Caflisch J. Locatelli I. Rousson V. Jenni O. G. (2012). Improvement in gross motor performance between 3 and 5 years of age. *Percept. Mot. Skills* 114 795–806. 10.2466/10.13.25.Pms.114.3.795-806 22913021

[B34] Klenberg L. Korkman M. Lahti-Nuuttila P. (2001). Differential development of attention and executive functions in 3- to 12-year-old finnish children. *Dev. Neuropsychol.* 20 407–428. 10.1207/S15326942DN2001_6 11827096

[B35] Korkman M. Kirk U. Kemp S. (1998). *NEPSY: A Developmental Neuropsychological Assessment.* San Antonio, TX: Pearson Assessment.

[B36] Kuhn M. Wing J. Weston S. Williams A. Keefer C. Engelhardt A. (2016). *Caret: Classification and Regression Training.* Available at: http://cran.r-project.org/package=caret

[B37] Lawson G. M. Duda J. T. Avants B. B. Wu J. Farah M. J. (2013). Associations between children’s socioeconomic status and prefrontal cortical thickness. *Dev. Sci.* 16 641–652. 10.1111/desc.12096 24033570 PMC3775298

[B38] Leeger-Aschmann C. S. Schmutz E. A. Radtke T. Kakebeeke T. H. Zysset A. E. Messerli-Burgy N. (2016). Regional sociocultural differences as important correlate of physical activity and sedentary behaviour in Swiss preschool children. *Swiss Med. Wkly.* 146:w14377. 10.4414/smw.2016.14377 27878789

[B39] Lim S. Han C. E. Uhlhaas P. J. Kaiser M. (2015). Preferential detachment during human brain development: age- and sex-specific structural connectivity in diffusion tensor imaging (DTI) data. *Cereb. Cortex* 25 1477–1489. 10.1093/cercor/bht333 24343892 PMC4428296

[B40] Livesey D. Keen J. Rouse J. White F. (2006). The relationship between measures of executive function, motor performance and externalising behaviour in 5-and 6-year-old children. *Hum. Mov. Sci.* 25 50–64. 10.1016/j.humov.2005.10.008 16442172

[B41] Lohman T. G. Roche A. F. Martorell R. (1988). *Anthropometric Standardization Reference Manual.* Champaign, IL: Human Kinetics Books.

[B42] Lupien S. J. Maheu F. Tu M. Fiocco A. Schramek T. E. (2007). The effects of stress and stress hormones on human cognition: Implications for the field of brain and cognition. *Brain Cogn.* 65 209–237. 10.1016/j.bandc.2007.02.007 17466428

[B43] Lupien S. J. McEwen B. S. Gunnar M. R. Heim C. (2009). Effects of stress throughout the lifespan on the brain, behaviour and cognition. *Nat. Rev. Neurosci.* 10 434–445. 10.1038/Nrn2639 19401723

[B44] McAlister A. Peterson C. C. (2006). Mental playmates: siblings, executive functioning and theory of mind. *Br. J. Dev. Psychol.* 24 733–751. 10.1348/026151005X70094

[B45] Messerli-Burgy N. Kakebeeke T. H. Arhab A. Stulb K. Zysset A. E. Leeger-Aschmann C. S. (2016). The Swiss Preschoolers’ health study (SPLASHY): objectives and design of a prospective multi-site cohort study assessing psychological and physiological health in young children. *BMC Pediatr.* 16:85. 10.1186/s12887-016-0617-7 27390933 PMC4939002

[B46] Meyer C. Hagmann-von Arx P. Lemola S. Grob A. (2010). Correspondence between the general ability to discriminate sensory stimuli and general intelligence. *J. Individ. Dif.* 31 46–56. 10.1027/1614-0001/a000006

[B47] Miyake A. Friedman N. (2012). The nature and organization of individual differences in executive functions: four general conclusions. *Curr. Dir. Psychol. Sci.* 21 8–14. 10.1177/0963721411429458 22773897 PMC3388901

[B48] Miyake A. Friedman N. P. Emerson M. J. Witzki A. H. Howerter A. Wager T. D. (2000). The unity and diversity of executive functions and their contributions to complex ”Frontal Lobe” tasks: a latent variable analysis. *Cogn. Psychol.* 41 49–100. 10.1006/cogp.1999.0734 10945922

[B49] Monti J. M. Hillman C. H. Cohen N. J. (2012). Aerobic fitness enhances relational memory in preadolescent children: the FITKids randomized control trial. *Hippocampus* 22 1876–1882. 10.1002/hipo.22023 22522428 PMC3404196

[B50] Moriguchi Y. (2014). The early development of executive function and its relation to social interaction: a brief review. *Front. Psychol.* 5:388. 10.3389/fpsyg.2014.00388 24808885 PMC4010730

[B51] Mulder H. Verhagen J. Van der Ven S. H. G. Slot P. L. Leseman P. P. M. (2017). Early executive function at age two predicts emergent mathematics and literacy at age five. *Front. Psychol.* 8:1706. 10.3389/fpsyg.2017.01706 29075209 PMC5643463

[B52] Niederer I. Kriemler S. Gut J. Hartmann T. Schindler C. Barral J. (2011). Relationship of aerobic fitness and motor skills with memory and attention in preschoolers (Ballabeina): a cross-sectional and longitudinal study. *BMC Pediatr.* 11:34. 10.1186/1471-2431-11-34 21569343 PMC3107157

[B53] Noble K. Norman M. F. Farah M. (2005). Neurocognitive correlates of socioeconomic status in kindergarten children. *Dev. Sci.* 8 74–87. 10.1111/j.1467-7687.2005.00394.x 15647068

[B54] Pate R. R. Almeida M. J. McIver K. L. Pfeiffer K. A. Dowda M. (2006). Validation and calibration of an accelerometer in preschool children. *Obesity* 14 2000–2006. 10.1038/oby.2006.234 17135617

[B55] Ploughman M. (2008). Exercise is brain food: the effects of physical activity on cognitive function. *Dev. Neurorehabil.* 11 236–240. 10.1080/17518420801997007 18781504

[B56] Reichle B. Franiek S. (2009). Erziehungsstil aus elternsicht - deutsche erweiterte version des alabama parenting questionnaire für grundschulkinder (DEAPQ-EL-GS). *Z. Entwicklungspsychol. Pädagog. Psychol.* 41 12–25. 10.1026/0049-8637.41.1.12

[B57] Roebers C. M. Kauer M. (2009). Motor and cognitive control in a normative sample of 7-year-olds. *Dev. Sci.* 12 175–181. 10.1111/j.1467-7687.2008.00755.x 19120425

[B58] Roebers C. M. Rothlisberger M. Neuenschwander R. Cimeli P. Michel E. Jager K. (2014). The relation between cognitive and motor performance and their relevance for children’s transition to school: a latent variable approach. *Hum. Mov. Sci.* 33 284–297. 10.1016/j.humov.2013.08.011 24289983

[B59] Röthlisberger M. Neuenschwander R. Michel E. Roebers C. (2010). Exekutive funktionen: zugrundeliegende kognitive prozesse und deren korrelate bei kindern im späten vorschulalter. *Z. Entwicklungspsychol. Pädagog. Psychol.* 42 99–110. 10.1026/0049-8637/a000010

[B60] Sawyer A. C. P. Miller Lewis L. Searle A. Sawyer M. Lynch J. (2015). Is greater improvement in early self-regulation associated with fewer behavioral problems later in childhood? *Dev. Psychol.* 51 1740–1755. 10.1037/a0039829 26501724

[B61] Spearman C. (1904). ”General intelligence ” objectively determined and measured. *Am. J. Psychol.* 15 201–292. 10.2307/1412107

[B62] Stadelmann S. Perren S. Kölch M. Groeben M. Schmid M. (2010). Psychisch kranke und unbelastete Eltern. elterliche stressbelastung und psychische symptomatik der kinder. *Kindheit Entwicklung* 19 72–81. 10.13109/prkk.2016.65.4.249 27027216

[B63] van Buuren S. Groothuis-Oudshoorn K. (2011). Mice: multivariate imputation by chained equations in R. *J. Stat. Softw.* 45 1–67. 10.18637/jss.v045.i03

[B64] Vandenbroucke L. Spilt J. Verschueren K. Baeyens D. (2017). Keeping the spirits up: the effect of teachers’ and parents’ emotional support on children’s working memory performance. *Front. Psychol.* 8:512. 10.3389/fpsyg.2017.00512 28421026 PMC5378781

[B65] Wehrle F. M. Kaufmann L. Benz L. D. Huber R. O’Gorman R. L. Latal B. (2016). Very preterm adolescents show impaired performance with increasing demands in executive function tasks. *Early Hum. Dev.* 92 37–43. 10.1016/j.earlhumdev.2015.10.021 26651084

[B66] Wunsch K. Pfister R. Henning A. Aschersleben G. Weigelt M. (2016). No interrelation of motor planning and executive functions across young ages. *Front. Psychol.* 7:1031. 10.3389/fpsyg.2016.01031 27462285 PMC4940395

[B67] Young S. E. Friedman N. P. Miyake A. Willcutt E. G. Corley R. P. Haberstick B. C. (2009). Behavioral disinhibition: liability for externalizing spectrum disorders and its genetic and environmental relation to response inhibition across adolescence. *J. Abnorm. Psychol.* 118 117–130. 10.1037/a0014657 19222319 PMC2775710

