# Supplementary material for: Predictors of Executive Functions in Preschoolers: Findings From the SPLASHY Study
Source: Front Psychol. 2018 Oct 29;9:2060. doi: 10.3389/fpsyg.2018.02060 (PMC6216414; doi:10.3389/fpsyg.2018.02060)
Supplement: Supplementary file 1 [file Table_1.docx]

Supplementary Material

Predictors of Executive Functions in Preschoolers: Findings from the SPLASHY study

**Annina E. Zysset^1^, Tanja H. Kakebeeke^1,2^, Nadine Messerli-Bürgy^3,6^, Andrea H. Meyer^4^, Kerstin Stülb^3^, Claudia S. Leeger-Aschmann^5^, Einat A. Schmutz^5^, Amar Arhab^6^, Jardena J. Puder^6,†^, Susi Kriemler^5†^, Simone Munsch^3†^, Oskar G. Jenni^1,2†*^**

^1^ Child Development Center, University Children’s Hospital Zurich, Zurich, Switzerland

^2^ Children’s Research Center, University Children’s Hospital Zurich, Zurich, Switzerland

^3^ Department of Clinical Psychology and Psychotherapy, University of Fribourg, Fribourg, Switzerland

^4^ Department of Psychology, University of Basel, Basel, Switzerland

^5^ Epidemiology, Biostatistics and Prevention Institute, University of Zurich, Zurich, Switzerland

^6^ Obstetric service, Lausanne University Hospital, Lausanne, Switzerland

^†^ shared last authors (consortium)

*** Correspondence:**Oskar G. Jenni, M.D.

[Oskar.Jenni@kispi.uzh.ch](mailto:Oskar.Jenni@kispi.uzh.ch)

# Supplementary Figures and Tables

**Supplementary Table 1.** Single regression analyses of tested predictors of executive functions.

|  | β | adjusted R^2^ | p-value |
| --- | --- | --- | --- |
| **Individual factors** |  |  |  |
| **Demographic and biological variables** |  |  |  |
| Sex | 0.18 | 0.03 | .00 |
| SES | 0.23 | 0.05 | .00 |
| Born preterm | - 0.13 | 0.01 | .04 |
| Body fat | - 0.03 | - 0.00 | .63 |
| Fine motor skills | 0.37 | 0.13 | .00 |
| Pure motor | 0.16 | 0.02 | .01 |
| Associated movements | 0.17 | 0.03 | .00 |
| Moderate to vigorous PA | 0.03 | - 0.00 | .63 |
| **Psychological variables** |  |  |  |
| Hyperactivity/Inattention | - 0.15 | 0.02 | .01 |
| Peer Problems | 0.02 | - 0.00 | .75 |
| Prosocial Behavior | 0.01 | - 0.00 | .90 |
| Emotionality temperament | 0.02 | - 0.00 | .71 |
| Visual perception | 0.31 | 0.09 | .00 |
| EFs (T1) | 0.45 | 0.20 | .00 |
| **Interpersonal factors** |  |  |  |
| **Family** |  |  |  |
| Parenting stress | 0.08 | 0.00 | .14 |
| Positive parenting | - 0.03 | - 0.00 | .63 |
| Inconsistent parenting | 0.04 | - 0.00 | .42 |
| Siblings | 0.09 | 0.01 | .10 |
| Time outdoors | 0.06 | 0.00 | .25 |
| Halfdays in childcare | - 0.14 | 0.02 | .01 |

**
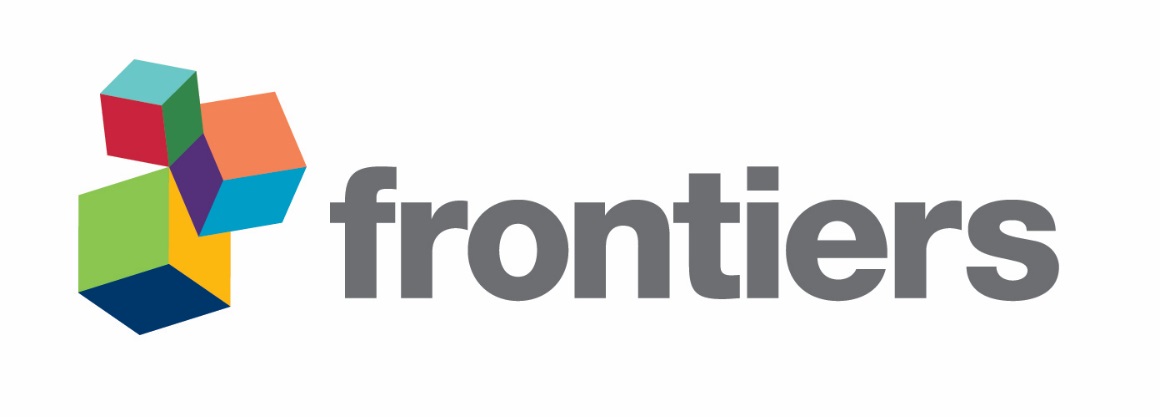
**
